# Cefadroxil as Oral Transitional Therapy for Gram-Positive Bacteremia With or Without Non-Vertebral Osteomyelitis: A Multicenter Retrospective Cohort Study

**DOI:** 10.1093/ofid/ofag413

**Published:** 2026-08-10

**Authors:** Devin C Clark, Rachel Baden, Josh Banerjee, Rita Bedrossian, Hannah Chute, Kusha Davar, Derek Deng, Matthew Donnelly, Daisy Fernandez, Reid Goodman, Hugh Gordon, Emerson Harris, Douglass Hutcheon, Arthur Jeng, Vaishnavi Kolluru, Pamela Lee, Esteban Martinez, Loren G Miller, Emi Minejima, Vadym Mykhaylov, Madelyn Perez, Dana Russell, Varsha Srinivasa, Ishaque Syed, Noah Wald-Dickler, Brad Spellberg, Sarah R Freling

**Affiliations:** Department of Medicine, Los Angeles General Medical Center, Los Angeles, California, USA; Department of Medicine, Keck School of Medicine of USC, Los Angeles, California, USA; Department of Medicine, Los Angeles General Medical Center, Los Angeles, California, USA; Department of Medicine, Keck School of Medicine of USC, Los Angeles, California, USA; Department of Medicine, Los Angeles General Medical Center, Los Angeles, California, USA; Department of Medicine, Keck School of Medicine of USC, Los Angeles, California, USA; Department of Medicine, Los Angeles General Medical Center, Los Angeles, California, USA; Department of Medicine, Keck School of Medicine of USC, Los Angeles, California, USA; Department of Medicine, Keck School of Medicine of USC, Los Angeles, California, USA; Department of Medicine, Los Angeles General Medical Center, Los Angeles, California, USA; Department of Medicine, Keck School of Medicine of USC, Los Angeles, California, USA; Department of Medicine, Los Angeles General Medical Center, Los Angeles, California, USA; Department of Medicine, Keck School of Medicine of USC, Los Angeles, California, USA; Department of Pharmacy, University of Southern California Mann School of Pharmacy and Pharmaceutical Sciences, Los Angeles, California, USA; Department of Medicine, Los Angeles General Medical Center, Los Angeles, California, USA; Department of Medicine, Keck School of Medicine of USC, Los Angeles, California, USA; Department of Medicine, Los Angeles General Medical Center, Los Angeles, California, USA; Department of Medicine, Keck School of Medicine of USC, Los Angeles, California, USA; Department of Medicine, Los Angeles General Medical Center, Los Angeles, California, USA; Department of Medicine, Keck School of Medicine of USC, Los Angeles, California, USA; Hospital Administration, Los Angeles General Medical Center, Los Angeles, California, USA; Department of Medicine, Keck School of Medicine of USC, Los Angeles, California, USA; Department of Medicine, Los Angeles General Medical Center, Los Angeles, California, USA; Department of Medicine, Keck School of Medicine of USC, Los Angeles, California, USA; Department of Medicine, Division of Infectious Diseases, Olive View-UCLA Medical Center, Sylmar, California, USA; Department of Medicine, University of California at Los Angeles, Los Angeles, California, USA; Department of Medicine, University of Southern California, Los Angeles, California, USA; Department of Medicine, Division of Infectious Diseases, Harbor-UCLA Medical Center, Torrance, California, USA; Department of Medicine, Los Angeles General Medical Center, Los Angeles, California, USA; Department of Medicine, Keck School of Medicine of USC, Los Angeles, California, USA; Department of Medicine, Division of Infectious Diseases, Harbor-UCLA Medical Center, Torrance, California, USA; Department of Pharmacy, University of Southern California Mann School of Pharmacy and Pharmaceutical Sciences, Los Angeles, California, USA; Department of Medicine, Los Angeles General Medical Center, Los Angeles, California, USA; Department of Medicine, Los Angeles General Medical Center, Los Angeles, California, USA; Department of Medicine, Keck School of Medicine of USC, Los Angeles, California, USA; Department of Medicine, Los Angeles General Medical Center, Los Angeles, California, USA; Department of Medicine, Keck School of Medicine of USC, Los Angeles, California, USA; Department of Medicine, Los Angeles General Medical Center, Los Angeles, California, USA; Department of Medicine, Keck School of Medicine of USC, Los Angeles, California, USA; Department of Medicine, Los Angeles General Medical Center, Los Angeles, California, USA; Department of Medicine, Keck School of Medicine of USC, Los Angeles, California, USA; Department of Medicine, Los Angeles General Medical Center, Los Angeles, California, USA; Department of Medicine, Keck School of Medicine of USC, Los Angeles, California, USA; Department of Medicine, Los Angeles General Medical Center, Los Angeles, California, USA; Department of Medicine, Keck School of Medicine of USC, Los Angeles, California, USA; Hospital Administration, Los Angeles General Medical Center, Los Angeles, California, USA; Department of Medicine, Los Angeles General Medical Center, Los Angeles, California, USA; Department of Medicine, Keck School of Medicine of USC, Los Angeles, California, USA

**Keywords:** bacteremia, cefadroxil, osteomyelitis, stewardship

## Abstract

**Background:**

Oral cefadroxil has favorable pharmacokinetics for the treatment of bloodstream infection (BSI) and osteomyelitis caused by Gram-positive cocci (GPC), but limited clinical data exist.

**Methods:**

We conducted a multicenter, retrospective cohort study between September 2022 and April 2025 assessing cefadroxil versus intravenous (IV)-only or other oral transitional therapy in adults with susceptible GPC BSI, with or without osteomyelitis. No patients with endocarditis, vertebral osteomyelitis, or prosthetic or central nervous system infections were included. Primary outcome was treatment success, defined as 90-day survival without infection progression or relapse. Prioritized secondary outcomes included all-cause mortality, recurrent bacteremia, and readmission at 90 days.

**Results:**

Of 323 patients with BSI, 58 were treated with cefadroxil, 72 IV only, and 193 with other oral antibiotics. Rates of osteomyelitis were comparable in each cohort, but skin and soft-tissue infections were a more common source of bacteremia in cefadroxil (52%) than either IV (28%, *P* = .01) or orally (PO) (31%, *P* = .003) cohorts. *Staphylococcus aureus* was more frequent in the IV (60%) than cefadroxil cohort (36%, *P* = .008). Nevertheless, cefadroxil-treated patients had a similar case mix index, expected mortality, and expected length of stay as the IV and PO cohorts, indicating comparable populations. Treatment success was similar for cefadroxil (91%), IV (94%, *P* = .51), and other oral (93%, *P* = .63) cohorts. Mortality, hospital readmission, and adverse events were comparable between groups.

**Conclusions:**

We found similar outcomes for cefadroxil and IV or PO alternatives in patients with GPC BSI, absent endocarditis, vertebral osteomyelitis, prosthetic or central nervous system infection. Cefadroxil was well-tolerated without observed severe adverse events.

**Main Point:**

In this multicenter, retrospective cohort study, cefadroxil was an effective and safe oral transitional therapy for bloodstream infections, with or without nonvertebral osteomyelitis.

## Graphical Abstract

**Graphical Abstract:**
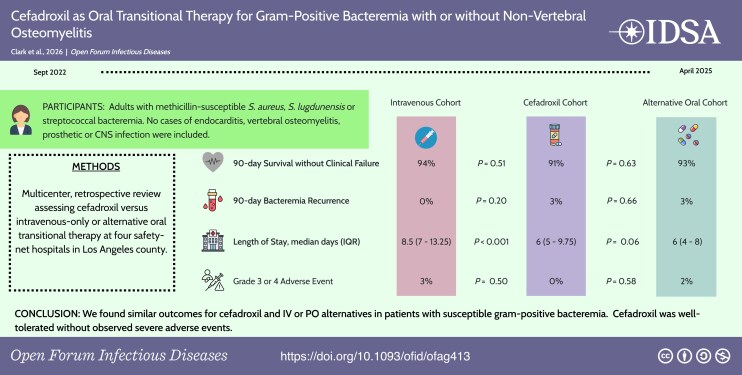
This graphical abstract is also available at Tidbit: https://tidbitapp.io/tidbits/cefadroxil-as-oral-transitional-therapy-for-gram-positive-bacteremia-with-or-without-non-vertebral-osteomyelitis?utm_campaign=tidbitlinkshare&amp;utm_source=ITP

Bloodstream infections (BSIs) are associated with significant morbidity, mortality, and healthcare utilization [1]. Gram-positive cocci (GPCs) account for approximately one half of all BSI pathogens in the United States [2], and often herald deep-seated infection, such as osteomyelitis. Historically, management of BSI and osteomyelitis has relied on prolonged intravenous (IV) therapy, but multiple recent randomized controlled trials (RCTs) and concordant large observational studies support transition to oral agents following initial stabilization [3–10]. Although parenteral β-lactams are the cornerstone of antibacterial therapy for BSIs and osteomyelitis, most studies have prioritized non–β-lactams for oral transitional therapy due to concerns about oral β-lactam bioavailability, serum concentrations, and pharmacokinetic/pharmacodynamic target attainment (ie, ability to achieve sufficient time above minimum inhibitory concentration, the primary driver of β-lactam efficacy). Nonetheless, oral β-lactams were included in the landmark OVIVA and POET randomized control trials evaluating osteomyelitis and endocarditis [6, 7], and an expanding body of evidence supports their use in both Gram-negative and Gram-positive BSI [11, 12].

Cefadroxil is an oral first-generation cephalosporin with activity and tolerability comparable to cephalexin [13, 14]. Approved by the Food and Drug Administration in 1978, it has active indications for skin and soft-tissue infection (SSTI), urinary tract infection, and pharyngitis/tonsillitis. It is principally clinically active against streptococcal species and methicillin-susceptible *Staphylococcus aureus* (MSSA) and thus has a narrower clinical antimicrobial spectrum than other agents, such as trimethoprim-sulfamethoxazole (TMP-SMX), fluoroquinolones, amoxicillin-clavulanate, and ceftriaxone. Nevertheless, pharmacokinetically, it has excellent bioavailability (>90%) and a longer half-life (∼1.5–2 hours), allowing for twice-daily dosing, in contrast to the 3- or 4-times-daily typically required for cephalexin for GPC infections [15]. It is also well-tolerated with an adverse-event rate of approximately 7% [14, 16].

Despite these favorable properties, cefadroxil is rarely used in the United States. For example, cephalexin was prescribed more than 25-fold more frequently among Medicare Part D beneficiaries in 2019 [17]. However, in recent years, there has been increased interest in cefadroxil [18–20], as evidenced by a 69% increase in prescriptions from 2019 to 2023 [17]. This interest is likely primarily driven by the desire to simplify regimens because more frequent dosing has been associated with decreased adherence and worse outcomes [21–23]. Although most studies on cefadroxil date from soon after its advent in the 1970s–1980 s, more recent studies have shown promising results for musculoskeletal infection [16, 24, 25], and it was among the most commonly used agents in 2 recent studies on oral transitional therapy [26, 27].

Following addition to the health system drug formulary in September 2022, cefadroxil has been increasingly used for susceptible invasive Gram-positive infections across Los Angeles County Department of Health Services (LAC DHS). We conducted a retrospective cohort study to assess clinical outcomes of patients with BSI, including cases with osteomyelitis as a nidus of infection, treated with cefadroxil versus either IV or alternative oral (PO) agents.

## METHODS

### Study Design

We analyzed all cases of BSI secondary to MSSA*, Staphylococcus lugdunensis,* and *Streptococcus* spp. susceptible to first-generation cephalosporins between 1 September 2022 and 30 April 2025 at the 4 LAC DHS hospitals: Harbor-University of California Los Angeles (Harbor-UCLA), Los Angeles General Medical Center (LAGMC), Olive View Medical Center, and Rancho Los Amigos. Other coagulase-negative *staphylococci* were not included because of a high rate of contamination and difficulty determining clinical significance.

### Patient Consent Statement

The University of Southern California Institutional Review Board approved this study with waiver of informed consent because the data were deidentified and followed the Strengthening the Reporting of Observational Studies in Epidemiology reporting guidelines [28].

### Patient Identification, Inclusion and Exclusion Criteria, and Definitions

Adult patients were identified by querying the common electronic medical record (EMR) database used by all 4 hospitals for blood cultures positive for included pathogens during the study period. All identified records were manually reviewed to determine eligibility. Included comparator patients received treatment with IV-only or alternative PO agents active against the infecting pathogen. Adjunctive antibiotics were allowed as long as they did not provide overlapping coverage of pathogen of interest. Exclusion criteria included blood culture organism deemed a contaminant by treating providers, discharge on multiple agents covering pathogen of interest (adjunctive rifampin excepted), discharge antibiotic not eligible for consideration (eg, long-acting lipoglycopeptide or agent not expected to have GPC activity), completed therapy before discharge (to prevent confounders as most patients receive IV therapy while admitted), end-stage renal disease on hemodialysis (because of ease of posthemodialysis IV therapy expected to skew groups), enrollment in a clinical trial, inadequate follow-up (less than 90 days from discharge), patient-directed discharge (“against medical advice”) without antibiotics, polymicrobial bacteremia with excluded pathogen, and transfer to an outside hospital. Following initial review, no patients with endocarditis, vertebral osteomyelitis, or prosthetic or central nervous system infection were identified in the cefadroxil cohort, so such cases were excluded from the IV and PO cohorts.

Clinical and demographic data to last known follow-up were extracted retrospectively from the LAC DHS Cerner EMR and the Vizient Clinical Data Base (CDB) [29]. For all inpatient encounters, case mix index, defined as the average of the relative weights of the Medicare Severity Diagnostic Related Groups that were assigned to patients at hospital discharge, and expected length of stay (LOS) and mortality, were extracted from the Vizient CDB. Expected LOS and mortality were calculated by the Vizient CDB using proprietary algorithms, based on patient age and the total sum of International Classification of Diseases-10CM and Current Procedural Technology codes. Stable housing was defined as residing in a personal or family residence, or medically adjacent support facility (eg, skilled nursing facility, recuperative housing) at time of treatment.

### Outcome Measures

The primary outcome was treatment success, defined as 90-day survival without infection progression or relapse. Prioritized secondary outcomes included all-cause mortality, recurrent bacteremia, and readmission at 90 days. Infection progression was defined as postdischarge clinical concern for worsening despite active antibiotics requiring unanticipated procedure. Relapse was defined as postdischarge recurrent infection with same site and pathogen after antibiotic completion. Hospital LOS was considered an outcome measure because discharge regimen is expected to directly impact LOS, consistent with multiple RCTs that have shown oral transitional therapy is associated with shorter LOS compared with long-term IV therapy [30].

We collected adverse events (AEs) of the following categories: gastrointestinal symptoms, cytopenias, metabolic and electrolyte abnormalities, hepatotoxicity, neuropathy, line complication, and drug allergy. We limited ourselves to these AEs as they were likely antibiotic-related effects rather than nonspecific symptoms such as fatigue, which could be from underlying diseases or from the drugs. Severity was defined by Common Terminology Criteria for Adverse Events, version 5.0 [31].

### Statistical Analysis

Cohort differences of categorical variables were examined using the χ^2^ test or Fisher exact test as appropriate. Continuous variables were analyzed pair-wise using the Mann-Whitney *U* test, or the Steel-Dwass test for nonparametric multiple comparisons adjustment. Initial analysis was performed using KyPlot statistical software, version 6.0 (KyensLab Inc). Multivariable Firth penalized logistic regression analysis of treatment arm and pathogen (*S aureus* vs other organisms) as independent predictors of clinical success was performed using Stata version 18 (StataCorp).

## RESULTS

### Study Population

**Figure 1.**
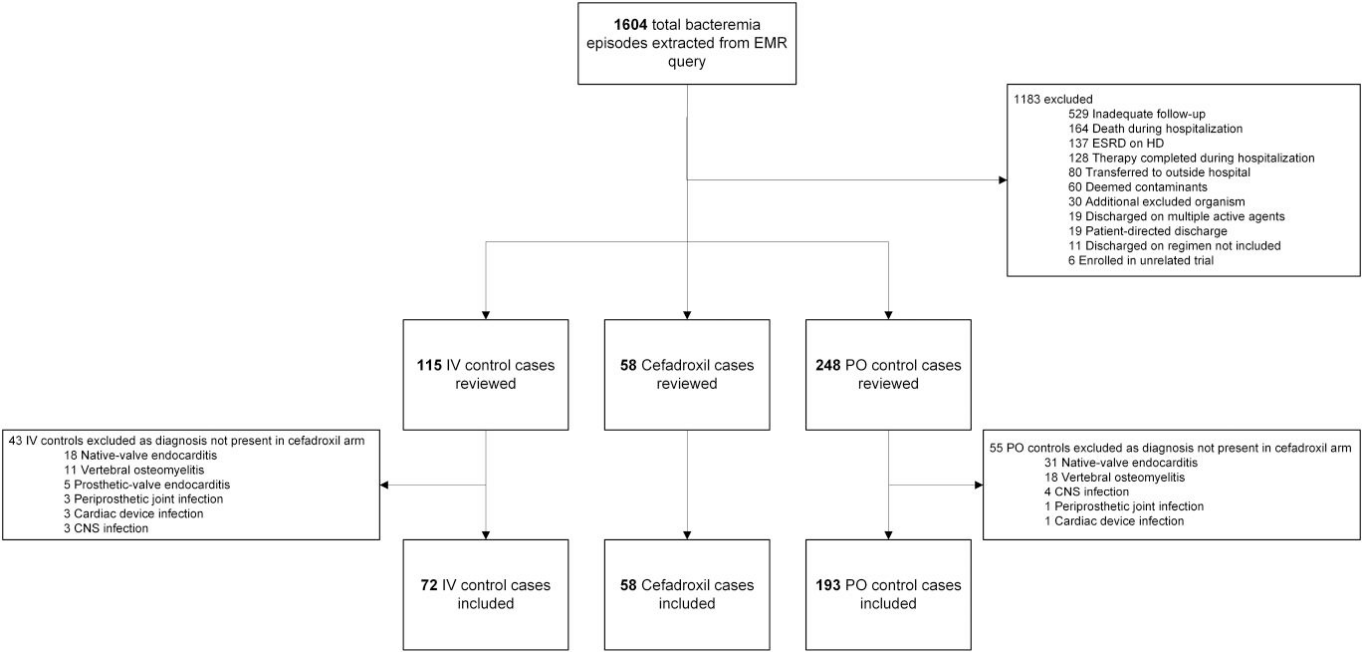
Screening and exclusion process.

The initial EMR query elicited 1604 total cases of bacteremia with included organisms. After chart review, 1183 met prespecified exclusion criteria, leaving 421 cases. Of these, 58 were treated with cefadroxil, 115 with IV antibiotics, and 248 with other PO antibiotics (Figure 1).

During chart review, we excluded 43 additional patients from the IV cohort because they had a nidus of infection that was not present in the cefadroxil-treated cohort (18 native-valve endocarditis, 11 vertebral osteomyelitis, 5 prosthetic-valve endocarditis, 3 periprosthetic joint infections [PJI], 3 cardiac device infections, and 3 central nervous system [CNS] infections); 55 patients were similarly excluded from the PO cohort (31 native-valve endocarditis, 18 vertebral osteomyelitis, 4 CNS infections, 1 PJI, 1 cardiac device infection). Ultimately, a total of 323 episodes were included: 58 cefadroxil, 72 IV cohort, and 193 PO cohort.

Baseline characteristics showed similar age and sex distribution in all groups (Table 1). Hispanic/Latino race was more common in the IV cohort with 82% versus 62% in cefadroxil group (*P* = .01). Comorbidities were similar aside from diabetes mellitus, malignancy, immunosuppressing medications, and substance use. When compared with the cefadroxil arm, diabetes mellitus was more common in the IV cohort (63% vs 45%, *P* = .04), malignancy more common in the PO cohort (14% vs 3%, *P* = .03); immunosuppressive medications more frequent in the IV cohort (15% vs 3%, *P* = .001); and polysubstance use much less common in the IV cohort (6% vs 28%, *P* = .001), but the PO cohort was similar (24%, *P* = .56). Similarly, housing instability was much more common in the cefadroxil group (28%) compared with the IV cohort (8%, *P* < .001), but similar to the PO cohort (20%, *P* = .23). Admission hospital differed significantly across the cohorts, with LA General representing the majority of cefadroxil-treated patients. Case mix index, expected LOS, and expected mortality were similar in all groups.

**Table 1.** Baseline Characteristics of Patients With Gram-Positive Bacteremia

…	Cefadroxil n = 58	IV Cohort n = 72		PO Cohort n = 193	
	n (%)	n (%)	*P* value	n (%)	*P* value
Age, median (IQR)	54 (48.25–59.75)	53.5 (44.75–59.25)	.64	52 (40–63)	.39
Sex, male (%)	44 (76%)	54 (75%)	.91	134 (69%)	.34
Race	…				
Asian	3 (5%)	1 (1%)	.32	4 (2%)	.20
Black	4 (7%)	3 (4%)	.70	28 (15%)	.18
Hispanic/Latino	36 (62%)	59 (82%)	.01	135 (70%)	.26
White	3 (5%)	1 (1%)	.32	5 (3%)	.39
Not Specified/Other	12 (21%)	7 (10%)	.08	21 (11%)	.05
Diabetes mellitus	26 (45%)	45 (63%)	.04	84 (44%)	.86
Cirrhosis	9 (16%)	10 (14%)	.79	24 (12%)	.54
Chronic Kidney Disease	10 (17%)	8 (11%)	.31	29 (15%)	.68
Malignancy	2 (3%)	7 (10%)	.30	27 (14%)	.03
Immunosuppressing Medications	2 (3%)	11 (15%)	.04	19 (10%)	.18
Polysubstance Use	16 (28%)	4 (6%)	.001	46 (24%)	.56
Injection Drug Use	4 (7%)	1 (1%)	.17	4 (2%)	.09
Valvular Disease	8 (14%)	5 (7%)	.08	20 (10%)	.47
Prosthetic material	3 (5%)	3 (4%)	>.99	16 (8%)	.57
Endovascular	0 (0%)	2 (3%)	.50	8 (4%)	.20
Housing Instability	16 (28%)	6 (8%)	<.001	39 (20%)	.23
Case mix index, median (IQR)	1.7 (1.0–2.0)	2.0 (1.1–2.5)	.07	2.0 (1.0–2.0)	.66
Expected LOS, median (IQR)	5.7 (4.5–8.4)	5.7 (4.8–9.4)	.86	5.5 (4.5–7.3)	.76
Expected mortality, median (IQR)	0.95% (0.002–1.9%)	1.3% (0.006–2.3%)	.52	1.0% (0.003–2.0%)	>.99
Admission Hospital	…				
LAGMC	47 (81%)	3 (4%)	<.001	132 (68%)	.06
Harbor-UCLA	0 (0%)	47 (65%)	<.001	27 (14%)	.001
OVMC	11 (19%)	21 (29%)	.18	33 (17%)	.74
RLA	0	1 (1%)	>.99	1 (0.5%)	>.99
Source	…				
Intraabdominal infection	1 (2%)	7 (10%)	.07	11 (6%)	.31
Odontogenic/sinusitis	1 (2%)	1 (1%)	>.99	6 (3%)	>.99
Osteomyelitis, nonvertebral	11 (19%)	19 (26%)	.32	32 (17%)	.67
Pneumonia/empyema	4 (7%)	6 (8%)	>.99	37 (19%)	.03
Pyelonephritis	1 (2%)	5 (7%)	.22	2 (1%)	.55
Septic arthritis	3 (5%)	6 (8%)	.73	15 (8%)	.77
Septic Thrombophlebitis	1 (2%)	3 (4%)	.63	1 (0.5%)	.41
Skin and soft tissue infection	30 (52%)	20 (28%)	.01	59 (31%)	.003
Unknown	8 (14%)	9 (13%)	.83	39 (20%)	.27
Pathogen	…				
*S aureus*	21 (36%)	43 (60%)	.008	53 (28%)	.20
*S lugdunensis*	0	0	N/A	1 (0.5%)	>.99
*S agalactiae*	10 (17%)	13 (18%)	.90	23 (12%)	.29
*S dysgalactiae*	5 (9%)	2 (3%)	.24	10 (5%)	.33
*S pneumoniae*	3 (5%)	1 (1%)	.32	31 (16%)	.05
*S pyogenes*	10 (17%)	3 (4%)	.02	35 (18%)	.88
*S. anginosus* Group	5 (9%)	8 (11%)	.64	14 (7%)	.73
*S. bovis* Group	0 (0%)	1 (1%)	>.99	4 (2%)	.58
Other Viridans Strep	5 (9%)	2 (3%)	.24	23 (12%)	.34
Source control	…				
Amputation	5 (9%)	12 (17%)	.18	15 (8%)	>.99
I&D, drain or washout	21 (36%)	28 (39%)	.75	48 (25%)	.09
Other	2 (3%)	2 (3%)	>.99	2 (1%)	.23
None	30 (52%)	30 (42%)	.25	128 (66%)	.04

Abbreviations: LAGMC: Los Angeles General Medical Center, Harbor-UCLA: Harbor-University of California Los Angeles, OVMC: Olive View Medical Center, RLA: Rancho Los Amigos. I&D: incision and drainage. LOS: length of stay.

Infectious source was similar in all groups aside from SSTI and pneumonia. SSTI was more frequently represented in the cefadroxil group (52%) than either the IV cohort (28%, *P* = .01) or PO cohort (31%, *P* = .003). Pneumonia was more common in the PO cohort (19% vs 7%, *P* = .03). Specific pathogen was also similar aside from MSSA, which was more frequently seen in the IV cohort (60%) than cefadroxil (36%, *P* = .008). Rates of source control were comparable between groups (Table 2).

**Table 2.** Antimicrobial Therapy

	Cefadroxil n = 58	IV Cohort n = 72		PO Cohort n = 193	
	n (%)	n (%)	*P* value	n (%)	*P* value
**Inpatient**	…				
Inpatient IV days, median (IQR)	5 (4–8)	8 (6.75–12)	<.001	5 (3–7)	.22
Inpatient switch to PO	9 (16%)	N/A		27 (14%)	.09
Inpatient PO days, median (IQR)	5 (2–6)	N/A		2 (1–3)	.06
**Discharge**	…				
*IV antibiotics*	…				
Ampicillin-sulbactam	…	1 (1%)			
Cefazolin	…	37 (51%)			
Ceftriaxone	…	26 (36%)			
Oxacillin	…	8 (11%)			
*Oral antibiotics*	…				
Amoxicillin	…	…		23 (12%)	
Amoxicillin-clavulanate	…	…		36 (19%)	
Cefadroxil	58 (100%)	…		…	
Cefdinir	…	…		5 (3%)	
Cephalexin	…	…		3 (2%)	
Clindamycin	…	…		4 (2%)	
Dicloxacillin	…	…		…	
Levofloxacin	…	…		36 (19%)	
Linezolid	…	…		60 (31%)	
Moxifloxacin	…	…		1 (0.5%)	
Penicillin	…	…		2 (1%)	
Trimethoprim-sulfamethoxazole	…	…		22 (11%)	
*Adjunctive antibiotics*	…				
Rifampin	1 (2%)	0	.45	8 (4%)	.69
Metronidazole	2 (3%)	5 (7%)	.46	7 (4%)	>.99
Other	1 (2%)	1 (1%)	>.99	4 (2%)	>.99
**Duration**	…				
Days of discharge regimen, median (IQR)	10 (7–14)	13 (7–25)	.25	10 (7–14)	.77
Completed discharge regimen	51 (88%)	57 (79%)	.19	175 (91%)	.54
Completed different regimen	7 (12%)	14 (19%)	.26	11 (6%)	.10
Did not complete antibiotics	0	1 (1%)	>.99	4 (2%)	.58
Total antibiotic days, median (IQR)	16 (14–29)	28.5 (16–44.25)	.004	15 (12–28)	.16
Abbreviation: IQR, interquartile.					

Discharge antibiotic in the IV cohort was most frequently cefazolin (51%), followed by ceftriaxone (36%) and oxacillin (11%). In the PO cohort, linezolid was most common (31%), followed by levofloxacin (19%), amoxicillin-clavulanate (19%), amoxicillin (12%), and TMP-SMX (11%). There was no difference in rates of adjunctive antibiotic use. The arms had similar completion rates and days of postdischarge regimen. However, the IV cohort had significantly longer median total duration of antibiotics at 28.5 days (interquartile range [IQR] 16–44.25) compared with 16 [IQR 14–29] in the cefadroxil group (*P* = .004). In the cefadroxil arm, >80% were prescribed 1 g twice-daily (or renally dosed equivalent).

### Outcomes

Clinical success was similar in all groups at 91% in the cefadroxil group, 94% (*P* = .51) in the IV cohort, and 93% (*P* = .63) in the PO cohort (Table 3). Treatment cohort was not found to be an independent predictor on multivariable analysis, but *S aureus* was associated with increased risk of failure (odds ratio 2.44; 95% confidence interval, 1.01–5.88) (Table 4).

**Table 3.** Outcomes

	Cefadroxil n = 58	IV Cohort n = 72		PO Cohort n = 193	
	n (%)	n (%)	P value	n (%)	P value
Treatment Success 90-day survival without clinical failure	53 (91%)	68 (94%)	.51	180 (93%)	.63
90-day mortality	1 (2%)	1 (1%)	>.99	1 (0.5%)	.41
LOS (days), median (IQR)	6 (5–9.75)	8.5 (7–13.25)	<.001	6 (4–8)	.06
LOS index, median (IQR)	0.8 (0.5–1.4)	1.2 (0.8–1.9)	.009	0.8 (0.5–1.2)	.56
Type of clinical failure	…				
Relapsed infection	3 (5%)	2 (3%)		9 (5%)	
Unplanned procedure	2 (3%)	1 (1%)		3 (2%)	
Suspected cause of failure	…				
Inadequate source control	3 (5%)	4 (6%)		5 (3%)	
Nonadherence	0	0		2 (1%)	
Underdosing	0	0		1 (0.5%)	
Unidentified source	2 (3%)	0		4 (2%)	
30-day bacteremia recurrence	0	0	>.99	1 (0.5%)	>.99
90-day bacteremia recurrence	2 (3%)	0	.20	5 (3%)	.66
90-day readmission	24 (41%)	23 (32%)	.27	63 (33%)	.36
Related to primary infection	11 (46%)	10 (44%)		31 (49%)	
Adverse events (grade 3 or 4)	0	2 (3%)	.50	4 (2%)	.58
Acute kidney injury	…	1 (1%)			
Anemia	…	1 (1%)		1 (0.5%)	
Hyperkalemia	…	…		2 (1%)	
Hyponatremia	…	1 (1%)			
Leukopenia	…	…		1 (0.5%)	
Transaminitis	…	2 (3%)			
Abbreviations: IQR, interquartile; LOS, length of stay.					

**Table 4.** Multivariable Analysis Assessing Odds of Treatment Failure

	Adjusted Odds Ratio	
	Estimate	95% Confidence Interval
Treatment arm	…	…
Cefadroxil	1.00	…
IV cohort	0.52	0.14–1.93
PO cohort	0.79	0.28–2.26
Pathogen	…	…
Non-*S aureus*	1.00	…
*S aureus*	2.44	1.01–5.88

The 5 cefadroxil failures comprised 3 relapsed infections and 2 unplanned procedures. Three of these cases appeared to have inadequate source control, and 2 had an unidentified source. The 1 death in the cefadroxil arm was due to a case of initially undiagnosed endocarditis in a patient with BSI caused by *Streptococcus agalactiae* endocarditis, which was treated with only a 7-day course of therapy. The 3 IV cohort failures related to infection were made up of 2 relapses and 1 unplanned procedure, all attributed to inadequate source control. The 12 PO cohort failures were 9 relapses and 3 unplanned procedures—5 attributed to inadequate source control, 4 to unidentified source, 2 to nonadherence, and 1 to underdosing. One death unrelated to infection occurred in both the IV and PO cohorts. Rates of recurrent bacteremia or readmission were comparable. Severe grade 3 or 4 AEs were similar in all arms (Table 3).

The IV cohort had a longer median LOS at 8.5 days (IQR 7–13.25) compared with 6 days (IQR 5–9.75) in the cefadroxil group (*P* < .001), with associated higher LOS index (indicating longer observed LOS relative to expected) and longer median inpatient IV course (8 [IQR 6.75–12] vs 5 [IQR 4–8] days, *P* < .001). The PO group had a similar LOS, duration of inpatient IV therapy, and frequency of inpatient oral transition (Table 3).

Subset analyses were performed for 2 higher risk populations of interest: *S aureus bacteremia* (SAB) and bone and joint infection. Among cases of SAB, there was no difference in infectious source or source control. The IV cohort received longer median inpatient antibiotics (9 days [IQR 7–13.5] vs 6 days [IQR 4–9], *P* = .004), but the total antibiotic duration was not different (20 days [IQR 15–42] vs 32 days [18.5–45.5], *P* = .06). There was no observed difference in rates of treatment success, but cefadroxil had significantly shorter duration of hospitalization (6 [IQR 5–12] vs 10 [IQR 8–15.5] days, *P* = .02) (Supplementary Tables 1–3). For bone and joint infections, there was no significant difference in infectious source or source control, duration of antibiotics or outcomes (Supplementary Tables 4–6).

## DISCUSSION

In this multicenter retrospective cohort study, we observed no difference in treatment success among patients with susceptible GPC bacteremia, including patients who had osteomyelitis, treated with oral cefadroxil versus IV and alternative PO regimens, in absence of selected high-risk sources (eg, endocarditis, PJI, vertebral osteomyelitis, CNS infection). We observed 90-day mortality, recurrent bacteremia, and readmission rates that were comparable, and no severe AEs occurred in the cefadroxil arm.

When compared to IV therapy, cefadroxil use in this setting may be preferable, given the shorter LOS and, more importantly, the lack of need to insert and maintain a long-term catheter while monitoring for its inherent complications (eg, thrombosis, line migration, infections). Although outcomes with alternative oral therapies are similar, β-lactams have long been lauded for their tolerability and lack of drug–drug interactions. In contrast, other oral options have well-known associated limitations. For example, linezolid can cause cytopenias or neuropathies with longer use and may interact with serotonergic medications. TMP-SMX use is challenging in renal dysfunction, causes hyperkalemia, and has higher hypersensitivity rates. Fluoroquinolones have multiple black-box warnings, including tendinopathies, and in the case of *S aureus* require adjunctive rifampin with associated hepatotoxicity and significant drug–drug interactions. Clindamycin is hampered by high rates of resistance, relatively poor gastrointestinal tolerability and propensity to cause *Clostridioides difficile* colitis. As mentioned, the historic limitation with β-lactams has been their more frequent dosing, but this is not an issue with twice-daily cefadroxil. Taken together, these factors make cefadroxil a favorable option among the oral antibiotics used in the treatment of BSI.

Although baseline characteristics were comparable in all groups (including case mix index, expected LOS, and expected mortality), several important differences between study arms warrant highlighting. From a demographic perspective, the most striking difference was the higher rate of polysubstance use and housing instability among patients receiving cefadroxil compared with IV cohorts. These differences partially reflect underlying variation in patient composition and practice patterns across study sites—the majority of cefadroxil cases were managed at LAGMC, which serves downtown Los Angeles with a large population of unhoused individuals with high rates of substance use. Additionally, housing instability presents a barrier to IV therapy because it often requires temporary housing or placement in a medical facility, which may not be an option due to personal preferences or socioeconomic pressures. As such, oral therapy may be preferentially used in this population.

Infectious source represents another potential confounder, along with associated longer duration of antibiotics in the IV arm. Although patients with selected high-risk sources were excluded from this analysis to address initial imbalances, a higher proportion of SSTI remained in the cefadroxil arm compared with both IV and PO cohorts. As SSTI is generally considered a lower risk source, this discrepancy might be expected to skew outcomes mor favorably in the cefadroxil arm. This distribution likely reflects institutional hesitancy to use cefadroxil for higher-risk infections in the absence of robust supporting data. Nevertheless, the cefadroxil cohort had similar case mix index, expected LOS, and expected mortality as the other cohorts, indicating patients with similar disease severity. Reassuringly, the subset analysis of bone and joint infections showed no significant differences in outcomes. However, analysis in larger cohorts and adjustments for differences in baseline patient characteristics, if any, would be helpful to confirm our findings.

The prevalence of SAB was higher in the IV cohort, likely reflecting current guidelines favoring IV therapy for all forms of SAB [32]. Given known association with increased risk of relapse and treatment failure [33], lower rates of SAB in the cefadroxil group could contribute to more favorable aggregate outcomes. However, the highest risk SAB syndromes (eg, endocarditis) were excluded, potentially narrowing expected outcome differences. Furthermore, subset analysis restricted to SAB found no observed difference in treatment success, despite a shorter duration of inpatient IV therapy in cefadroxil arm. Again, analysis in larger cohorts using adjustments for patient characteristics would be helpful to confirm our findings.

### Limitations

The primary limitation is the retrospective observational design, which introduces susceptibility to unmeasured confounding and selection bias. Additionally, baseline imbalances must be considered when interpreting results. Although hospital-specific practice patterns may have provided a partial mitigating, quasi-randomizing effect, residual confounding cannot be excluded. Importantly, this study excluded select high-risk sources, such as endocarditis and vertebral osteomyelitis, so findings should not be extrapolated to these disease entities. Last, patients in both oral groups received a median of 5 days IV lead-in therapy, which may have been sufficient for some simple non-MSSA infections and leaves effectiveness of earlier oral transition unevaluated. However, we emphasize given the uncertainty of the necessity of the last several days of therapy, use of a regimen with the lowest risk of adverse events is logical and preferred, and thus cefadroxil would be preferred to IV regimens in this case.

### Future Directions

Our findings support use of cefadroxil in future RCTs assessing oral transitional regimens for BSIs—in fact, it is an included option in the forthcoming *S aureus* Network Adaptive Platform trial assessing early oral switch therapy [34]. Large observational studies with infectious sources excluded in our study (eg, endocarditis, vertebral osteomyelitis) would also be of great interest. Additional data on specific infections (eg, osteomyelitis) would also be helpful. From a pharmacology perspective, additional data are needed to inform optimal cefadroxil dosing strategies, particularly for invasive infections, patients with obesity, and those with altered pharmacokinetics.

## CONCLUSION

In this multicenter retrospective cohort study, cefadroxil as transitional therapy was associated with similar treatment success, mortality and recurrence rates compared with IV and alternative oral antibiotics for patients with MSSA and streptococcal BSI, excluding selected high-risk sources. Cefadroxil was well-tolerated with no severe adverse events observed. Our findings support the use of cefadroxil as a pragmatic option for transitional therapy in lower risk infections.

## Supplementary Material

ofag413_Supplementary_Data

## Supplementary Data

Supplementary materials are available at *Open Forum Infectious Diseases* online. Consisting of data provided by the authors to benefit the reader, the posted materials are not copyedited and are the sole responsibility of the authors, so questions or comments should be addressed to the corresponding author.
