# Supplementary material for: Cefadroxil as Oral Transitional Therapy for Gram-Positive Bacteremia With or Without Non-Vertebral Osteomyelitis: A Multicenter Retrospective Cohort Study
Source: Open Forum Infect Dis. 2026 Aug 10;13(8):ofag413. doi: 10.1093/ofid/ofag413 (PMC13455355; doi:10.1093/ofid/ofag413)
Supplement: Supplementary file 1 [file ofag413_supplementary_data.docx]

**Table of Contents**

Table 1 *Staphylococcus aureus* Infection Characteristics………………………….…………….…………….	2

Table 2: *Staphylococcus aureus* Antimicrobial Therapy ……………………….……………………………...	3

Table 3. *Staphylococcus aureus* Outcomes ……….……………………………………...................................	4

Table 4. Bone and Joint Infection Characteristics ……….…………………………….....................................	5

Table 5. Bone and Joint Antimicrobial Therapy ……….…………………………….......................................	6

Table 4. Bone and Joint Outcomes ……….…………………………….................................…………………	7

**Table 1. *Staphylococcus aureus* Infection Characteristics**

|  | Cefadroxil  n=21 | IV Cohort  n=43 | | PO Cohort  n=53 | |
| --- | --- | --- | --- | --- | --- |
|  | n (%) | n (%) | *P* value | n (%) | *P* value |
| Source |  | | | | |
| Intraabdominal infection | 0 (0%) | 2 (5%) | >.99 | 5 (9%) | 0.31 |
| Osteomyelitis, nonvertebral | 6 (29%) | 15 (35%) | 0.61 | 12 (23%) | 0.59 |
| Pneumonia/empyema | 0 (0%) | 5 (12%) | 0.16 | 4 (8%) | 0.57 |
| Pyelonephritis | 0 (0%) | 1 (2%) | >.99 | 1 (2%) | >.99 |
| Septic arthritis | 2 (10%) | 5 (12%) | >.99 | 10 (19%) | 0.49 |
| Septic Thrombophlebitis | 1 (5%) | 2 (5%) | >.99 | 0 (0%) | 0.28 |
| Skin and soft tissue infection | 10 (48%) | 14 (33%) | 0.24 | 18 (34%) | 0.28 |
| Unknown | 2 (10%) | 2 (5%) | 0.59 | 9 (17%) | 0.72 |
| Source control |  | | | | |
| Amputation | 3 (14%) | 9 (21%) | 0.74 | 1 (2%) | 0.07 |
| I&D, drain or washout | 8 (38%) | 19 (44%) | 0.64 | 20 (38%) | 0.98 |
| None | 10 (48%) | 15 (35%) | 0.33 | 32 (60%) | 0.32 |
| I&D: incision and drainage | | | | | |

**Table 2. *Staphylococcus aureus* Antimicrobial Therapy**

|  | Cefadroxil  n=21 | IV Cohort  n=43 | | PO Cohort  n=53 | |
| --- | --- | --- | --- | --- | --- |
|  | n (%) | n (%) | *P* value | n (%) | *P* value |
| **Inpatient Antibiotics** |  | | | | |
| Inpatient IV Days, median (IQR) | 6 (4-9) | 9 (7-13.5) | 0.004 | 5 (4-8) | 0.52 |
| Inpatient switch to PO | 4 (19%) | N/A | | 11 (21%) | >.99 |
| Inpatient PO days, median (IQR) | 6.5 (4.75-7.25) | N/A | | 2 (1.5-3) | 0.15 |
| **Discharge** |  | | | | |
| *IV Antibiotics* |  | | | | |
| Cefazolin |  | 34 (81%) | |  | |
| Oxacillin |  | 8 (19%) | |  | |
| *Oral antibiotics* |  | | | | |
| Amoxicillin-clavulanate |  |  | | 1 (2%) | |
| Cefadroxil | 20 (100%) |  | |  | |
| Cefdinir |  |  | | 1 (2%) | |
| Cephalexin |  |  | | 1 (2%) | |
| Dicloxacillin |  |  | | 1 (2%) | |
| Levofloxacin |  |  | | 7 (13%) | |
| Linezolid |  |  | | 24 (45%) | |
| Trimethoprim-Sulfamethoxazole |  |  | | 17 (32%) | |
| *Adjunctive antibiotics* |  | | | | |
| Rifampin | 1 (5%) | 0 | 0.33 | 7 (13%) | 0.43 |
| Metronidazole | 1 (5%) | 2 (5%) | >.99 | 0 | 0.28 |
| Other | 1 (5%) | 1 (2%) | >.99 | 0 | 0.28 |
| **Duration** |  | | | | |
| Days of discharge regimen, median (IQR) | 10 (7-21) | 14 (7-25) | 0.48 | 13 (10-30) | 0.08 |
| Completed discharge regimen | 15 (71%) | 34 (79%) | 0.50 | 46 (87%) | 0.12 |
| Completed different regimen | 6 (29%) | 9 (21%) | 0.50 | 6 (11%) | 0.07 |
| Documented incomplete course | 0 | 0 | >.99 | 1 (2%) | >.99 |
| Total antibiotic days, median (IQR) | 20 (15-42) | 32 (18.5-45.5) | 0.06 | 21 (15-45) | 0.90 |
| IQR: Interquartile | | | | | |

**Table 3. *Staphylococcus aureus* Outcomes**

|  | Cefadroxil  n=21 | IV Cohort  n=43 | | PO Cohort  n=53 | |
| --- | --- | --- | --- | --- | --- |
|  | n (%) | n (%) | *P* value | n (%) | *P* value |
| Treatment Success  90-day survival without clinical failure | 20 (95%) | 40 (93%) | >.99 | 45 (85%) | 0.43 |
| 90-day Mortality | 0 (0%) | 0 (0%) | >.99 | 1 (2%) | >.99 |
| Length of stay (days), median (IQR) | 6 (5-12) | 10 (8-15.5) | 0.02 | 6 (4-9) | 0.35 |
| 30-day Bacteremia Recurrence | 0 | 0 | >.99 | 0 | >.99 |
| 90-day Bacteremia Recurrence | 0 | 0 | >.99 | 3 (6%) | 0.55 |
| 90-day Readmission | 8 (38%) | 14 (33%) | 0.66 | 20 (38%) | 0.98 |
| Related to Primary Infection | 3 (38%) | 7 (50%) | | 13 (65%) | |
| IQR: Interquartile | | | | | |

**Table 4. Bone and Joint Infection Characteristics**

|  | Cefadroxil  n=14 | IV Cohort  n=24 | | PO Cohort  n=42 | |
| --- | --- | --- | --- | --- | --- |
|  | n (%) | n (%) | *P* value | n (%) | *P* value |
| Pathogen |  | | | | |
| *S. aureus* | 8 (57%) | 19 (79%) | 0.15 | 18 (43%) | 0.35 |
| *S. agalactiae* | 3 (21%) | 5 (21%) | >.99 | 11 (26%) | >.99 |
| *S. dysgalactiae* | 1 (7%) | 0 (0%) | 0.37 | 0 (0%) | 0.25 |
| *S. pneumoniae* | 0 | 0 (0%) | >.99 | 2 (5%) | >.99 |
| *S. pyogenes* | 0 | 0 (0%) | >.99 | 4 (10%) | 0.56 |
| *S. anginosus* Group | 2 (14%) | 1 (4%) | 0.54 | 4 (10%) | 0.63 |
| Other Viridans Strep | 1 (7%) | 0 (0%) | >.99 | 3 (7%) | >.99 |
| Source control |  | | | | |
| Amputation | 3 (21%) | 11 (46%) | 0.18 | 9 (21%) | >.99 |
| I&D, drain or washout | 8 (57%) | 10 (42%) | 0.36 | 22 (52%) | 0.76 |
| None | 3 (21%) | 3 (13%) | 0.65 | 11 (26%) | >.99 |
| I&D: incision and drainage | | | | | |

**Table 5. Bone and Joint Antimicrobial Therapy**

|  | Cefadroxil  n=14 | IV Cohort  n=24 | | PO Cohort  n=42 | |
| --- | --- | --- | --- | --- | --- |
|  | n (%) | n (%) | *P* value | n (%) | *P* value |
| **Inpatient Antibiotics** |  | | | | |
| Inpatient IV Days, median (IQR) | 7.5 (5-19.25) | 8.5 (7-13.25) | 0.52 | 7.5 (5-12.75) | 0.54 |
| Inpatient switch to PO | 2 (14%) | N/A | | 7 (17%) | >.99 |
| Inpatient PO days, median (IQR) | 3.5 (2.25-4.75) | N/A | | 3 (2.5-3.75) | 0.88 |
| **Discharge** |  | | | | |
| *IV Antibiotics* |  | | | | |
| Ampicillin-Sulbactam |  | 1 (4%) | |  | |
| Cefazolin |  | 17 (71%) | |  | |
| Ceftriaxone |  | 3 (13%) | |  | |
| Oxacillin |  | 3 (13%) | |  | |
| *Oral antibiotics* |  | | | | |
| Amoxicillin |  |  | | 1 (2%) | |
| Amoxicillin-clavulanate |  |  | | 13 (31%) | |
| Cefadroxil | 14 (100%) |  | |  | |
| Clindamycin |  |  | | 3 (7%) | |
| Levofloxacin |  |  | | 7 (17%) | |
| Linezolid |  |  | | 7 (17%) | |
| Penicillin |  |  | | 1 (2%) | |
| Trimethoprim-Sulfamethoxazole |  |  | | 10 (24%) | |
| *Adjunctive antibiotics* |  | | | | |
| Rifampin | 0 | 0 | >.99 | 2 (5%) | >.99 |
| Metronidazole | 2 (14%) | 2 (8%) | 0.62 | 3 (7%) | 0.59 |
| **Duration** |  | | | | |
| Days of discharge regimen, median (IQR) | 17.5 (10-41.5) | 18 (7-31.25) | 0.43 | 19 (12.5-41.25) | 0.83 |
| Completed discharge regimen | 11 (79%) | 16 (67%) | 0.49 | 37 (88%) | 0.40 |
| Completed different regimen | 3 (21%) | 7 (29%) | 0.72 | 4 (10%) | 0.35 |
| Did not complete antibiotics | 0 | 1 (4%) | >.99 | 0 | >.99 |
| Total antibiotic days, median (IQR) | 47.5 (20.25-67.5) | 42.5 (21-46) | 0.36 | 42.5 (29.25-47) | 0.34 |
| IQR: Interquartile | | | | | |

**Table 6. Bone and Joint Outcomes**

|  | Cefadroxil  n=14 | IV Cohort  n=24 | | PO Cohort  n=42 | |
| --- | --- | --- | --- | --- | --- |
|  | n (%) | n (%) | *P* value | n (%) | *P* value |
| Treatment Success  90-day survival without clinical failure | 11 (79%) | 21 (88%) | 0.65 | 37 (88%) | 0.40 |
| 90-day Mortality | 0 | 1 (4%) | >.99 | 0 | >.99 |
| Length of stay (days), median (IQR) | 6.5 (5-17.75) | 9.5 (7-13.25) | 0.31 | 8.5 (6-13) | 0.96 |
| 90-day Bacteremia Recurrence | 0 | 0 | >.99 | 1 (2%) | >.99 |
| 90-day Readmission | 6 (43%) | 8 (33%) | 0.56 | 15 (36%) | 0.63 |
| Related to Primary Infection | 6 (100%) | 3 (38%) | | 11 (73%) | |
